# Laparoscopic Colectomy vs Laparoscopic CME: a Retrospective Study of Two Hospitals with Comparable Laparoscopic Experience

**DOI:** 10.1007/s11605-019-04502-8

**Published:** 2020-02-05

**Authors:** Juha KA Rinne, Anu Ehrlich, Jaana Ward, Ville Väyrynen, Mikael Laine, Ilmo H Kellokumpu, Matti Kairaluoma, Marja K Hyöty, Jyrki AO Kössi

**Affiliations:** 1 Päijät-Häme Central Hospital, Keskussairaalankatu 7, 15850 Lahti, Finland; 2 Tampere University, Tampere, Finland; 3 Department of Abdominal Surgery, Jorvi Hospital, Hospital District of Helsinki and Uusimaa, Turuntie 150, PL 800, 00029 HUS, Espoo, Finland; 4 Department of Gastrointerstinal Surgery, Central Hospital of Central Finland, Keskussairaalantie 19, 40620 Jyväskylä, Finland; 5 Department of Abdominal Surgery, Porvoo Hospital, Hospital District of Helsinki and Uusimaa, Porvoo, Finland; 6 Department of Gastroenterology, Tampere University Hospital, Teiskontie 35, 33520 Tampere, Finland

**Keywords:** Laparoscopic, Colon, Cancer, CME, NCME

## Abstract

**Purpose:**

To compare laparoscopic non-CME colectomy with laparoscopic CME colectomy in two hospitals with similar experience in laparoscopic colorectal surgery.

**Methods:**

Data was collected retrospectively from Päijät-Häme Central Hospital (PHCH, NCME group) and Central Finland Central Hospital (CFCH, CME group) records. Elective laparoscopic resections performed during 2007–2016 for UICC stage I–III adenocarcinoma were included to assess differences in short-term outcome and survival.

**Results:**

There were 340 patients in the NCME group and 325 patients in the CME group. CME delivered longer specimens (*p* < 0.001), wider resection margins (*p* < 0.001), and more lymph nodes (*p* < 0.001) but did not result in better 5-year overall or cancer-specific survival (NCME 77.9% vs CME 72.9%, *p* = 0.528, NCME 93.2% vs CME 88.9%, *p* = 0.132, respectively). Thirty-day morbidity, mortality, and length of hospital stay were similar between the groups. Conversion to open surgery was associated with decreased survival.

**Discussion:**

Complete mesocolic excision (CME) is reported to improve survival. Most previous studies have compared open CME with open non-CME (NCME) or open CME with laparoscopic CME. NCME populations have been historical or heterogeneous, potentially causing bias in the interpretation of results. Studies comparing laparoscopic CME with laparoscopic NCME are few and involve only small numbers of patients. In this study, diligently performed laparoscopic non-CME D2 resection delivered disease-free survival results comparable with laparoscopic CME but was not safer.

## Introduction

Colorectal cancer is the second most common cancer in women and third most common in men, especially in the developed world.^1,2^ Despite advances in chemo- and immunotherapy, surgery is still the only potentially curative treatment for colon cancer. Mesocolic excision together with locoregional lymph node removal has long been employed in colon cancer surgery. Anatomically based colon surgery is performed along embryological planes to acquire sufficient margins of healthy tissue around the tumor, including the supplying vessels and lymph nodes en bloc without unnecessary manipulation and mutilation of the specimen.^3–5^

Hohenberger et al. introduced the concept of complete mesocolic excision (CME), which resulted in marked improvements in the quality of surgery, decreased local recurrence rate, and improved survival.^6,7^ Similar results have been reported by West ^8^ and Bertelsen.^9,10^ Recent reviews and other reports have also supported the view that CME results in better oncologic outcome.^11,12^ Most previous studies, however, have compared open CME with open non-CME (NCME) or with a mixture of open and laparoscopic operations. NCME populations have been historical or heterogeneous, potentially causing bias in the interpretation of results. There are few reports on laparoscopic and robotic CME in the literature,^13–16^ and the feasibility and benefits of laparoscopic CME are not well defined. We therefore compared laparoscopic CME with laparoscopic NCME performed in two hospitals with long experience in laparoscopic colorectal surgery. Our hypothesis was that CME surgery provides better long-term survival.

## Material and Methods

Päijät-Häme Central Hospital (PHCH) and Central Finland Central Hospital (CFCH) have over two decades of experience in laparoscopic colorectal surgery. Perioperative care and operative strategies are standardized at both hospitals. CFCH started to perform laparoscopic CME surgery in the early 2000s according to the principles set out by Hohenberger,^17^ while PHCH continued to perform laparoscopic NCME surgery following the traditional D2 (Japanese Society for Cancer of the Colon and Rectum (JSCCR)) plane of dissection.

### Patients and Data Acquisition

CFCH has published its surgical technique and results, which include patients with stage I–III colon cancer who underwent CME colectomy between 2003 and 2011.^18^ An update of CFCH records was performed to include all elective patients operated on using laparoscopic CME during 2007–2016, and excluding patients operated on during the implementation phase of CME. At PHCH, patients with a diagnosis of colorectal cancer (ICD-10) or with a code for variants of colectomy were obtained from the hospital database for the same time period. Benign diseases and other pathologies such as neuroendocrine tumors and tumors of the appendix were excluded. The search results were then cross-checked for duplicates. Excluded were patients undergoing open surgery, those with stage IV colon cancer, cancer occurring in the setting of inflammatory bowel disease, or an emergency situation such as obstruction and perforation.

Data was collected retrospectively from hospital records in structured format for analysis. The data was checked for heterogeneity and any outliers checked manually. Causes of death were obtained from the Finnish Population Register Center. Data sets were pooled for analysis with CFCH representing the CME arm and PHCH the NCME arm. The study was a retrospective register study and permission for data acquisition was received from both institutions. Because of the study design, according to Finnish law, ethics committee approval was not needed.

### Surgical Technique

#### Right Hemicolon

Both hospitals had similar perioperative care protocols. Low molecular weight heparin was administered preoperatively at CFCH and at 6 h postoperative at PHCH. No mechanical bowel preparation or oral antibiotics were used. Antibiotic prophylaxis (cephalosporin + metronidazole) was given intravenously 30–60 min preoperatively. Both hospitals used 12 mmHg insufflation and a 4–5 trocar technique. Dissection was performed medial to lateral following a plane between visceral (Told’s) and parietal (Gerota’s) fascia. The dissection line traversed the head of the pancreas but gastroepiploic or infrapyloric lymph nodes were not routinely dissected. At PHCH, the dissection line followed the superior mesenteric vein (SMV) keeping lateral to it in the D2 dissection plane, whereas at CFCH, dissection followed the principles described by Hohenberger ^6, 17^ removing fat covering the SMV and thus removing lymph nodes in the D3 zone. Ileocolic artery (ICA), right colic artery (RCA) (when present), and the right branch of middle colic (MCA) were taken according to the plane of dissection. CFCH aimed for a 10-cm longitudinal margin to remove all involved pericolic lymph nodes, whereas a minimum of 5 cm of healthy bowel was considered the lowest acceptable limit at PHCH. The type of anastomosis was left to the surgeon’s discretion.

#### Transverse Colon

At PHCH, tumors of the transverse colon were managed with segmental resection taking the trunk of the MCA at the level of the pancreas. A minimum of 5 cm of longitudinal margin was considered a resection with curative intent. RCA if present was not routinely dissected. CFCH performed extended right or left hemicolectomies in accordance with the CME principle, removing the corresponding arteries following the D3 plane. In carcinomas of the splenic flexure, the inferior mesenteric artery (IMA) was taken at CFCH. At PHCH, the left colic artery (LCA) was taken but IMA spared. Division of the inferior mesenteric vein (IMV) was performed at the level of the pancreas at both hospitals.

#### Left Hemicolon

Carcinomas of the descending and sigmoid colon were operated on in similar fashion at both hospitals. IMV was excised at the level of the pancreas. IMA was divided 1 to 2 cm from the aorta to preserve the hypogastric nerves. CFCH aimed for a 10-cm longitudinal margin, except in the upper rectum, where a 5-cm distal margin was deemed appropriate. PHCH accepted a 5-cm margin. Splenic flexure mobilization was optional. Anastomosis was performed with a circular stapler at both hospitals.

Conversion to open surgery was defined as the necessity to interrupt the laparoscopic procedure and to proceed using the conventional technique. This was commonly due to adhesions from previous operations, unexpected invasion of an adjacent organ, or in order to ensure the optimal quality of the dissection.

#### Pathological Examination

Histopathological examination was performed by staff pathologists according to the UICC TNM classification (ref. 7th ed.). The examination was carried out using fixed specimens. In addition to total number of lymph nodes, a lymph node ratio (LNR) was calculated from positive nodes/nodes examined using the following cutoff values: LNR I < 10%, LNR II 10–25%, and LNR III > 25%. The quality of the surgical specimen was not reported at either hospital due to the retrospective nature of this study.

#### Adjuvant Therapy

Stage III patients received adjuvant therapy with capecitabine (or 5-fluorouracil, 5-FU) combined with oxaliplatin for 6 months. Stage II patients with at least one risk factor (vascular or perineural invasion, invasion of lymphatic ducts) or fewer than 12 lymph nodes studied received adjuvant chemotherapy selectively.

#### Follow-up

At both hospitals, follow-up included carcinoembryonic antigen (CEA) level every 3 to 6 months for the first 3 years and annually thereafter up to 5 years or until death. At CFCH, ultrasonographic investigation of the liver and chest radiograph or CT was performed annually. At PHCH, no routine imaging studies were conducted if CEA levels were stable and the patient was symptom free. If there was a rising tendency in CEA level, even if still well within normal limits (< 5.0 μg/L), a full-body CT was performed. Colonoscopy was performed at 2 and 5 years at PHCH and at 5 years at CFCH. Further characterization of metastases was carried out using CT, magnetic resonance imaging (MRI), and CT-positron emission tomography (PET).

Locoregional recurrence was defined as a recurrent tumor at the anastomotic site, recurrence in regional lymph, nodes, or peritoneal spread in the abdomen; CT, MRI, and endoscopy were used to ascertain whether newly diagnosed distant metastases were absent or present. The date of last follow-up was defined as the date when the patient record was reviewed at the end of 2017 or the date of death.

#### Statistical Analysis

Comparisons of continuous variables were performed using Student’s *t* test and comparisons of categorical variables using the *χ*^2^ test. Results are given as mean (SD) or median (interquartile range, IQR). The Kaplan–Meier method was used to calculate survival and the differences between groups were compared using the log-rank test. Overall survival (OS) was calculated from the date of surgery to the date of death from any cause, and cancer-specific survival (CS) was calculated from the date of surgery until the time of death from colon cancer or the end of follow-up. Recurrence-free survival (RFS) was calculated from the date of surgery to the date of the first recurrence, whether local or distant. The endpoint of locoregional recurrence at 5-year follow-up was the date of first locoregional recurrence. All statistical tests were two-sided. A *p* value less than 0.05 was considered significant. Statistical Package for the Social Sciences (SPSS) version 25.0 for Windows (IBM SPSS Statistics 25.0, USA) was used for statistical analysis.

## Results

A total of 340 patients in the NCME group and 325 patients in the CME group were included. Baseline characteristics are shown in Table 1. Median age, gender, BMI, and tumor site distribution were similar in both study groups. There were more ASA 3–4 patients in the NCME group.

**Table 1:** Baseline characteristics and treatment

	CME	NCME	*p* value
Age	72 ± 11 (25–96)	72 ± 10 (38–95)	0.515
Gender			0.752
Male	170 (52.3%)	182 (53.5%)	
Female	155 (47.7%)	158 (46.5%)	
BMI, mean (range)	26.5 (16.9–42.6)	26.6 (16.5–42.6)	0.772
ASA			< 0.001
1–2	166 (51.5%)	122 (35.9%)	
3–4	151 (46.4%)	218 (64.1%)	
Tumor location			0.133
Right colon	168 (51.7%)	168 (49.4%)	
Transverse colon	23 (7.1%)	12 (3.5%)	
Left colon	133 (40.9%)	158 (46.5%)	
Type of operation			< 0.001
Right hemicolectomy	165 (50.8%)	172 (50.6%)	
Extended right hemicolectomy	29 (8.9%)	0 (0.0%)	
Resection of transverse colon	0 (0.0%)	10 (2.9%)	
Left hemicolectomy	31 (9.5%)	15 (4.4%)	
Sigmoid resection	81 (24.9%)	118 (34.7%)	
Anterior resection	14 (4.3%)	19 (5.6%)	
Adjuvant therapy			
Stage I	0	9 (14.5%)	0.007
Stage II	24 (17.5%)	75 (48.1%)	< 0.001
Stage III	99 (79.2%)	103 (83.7%)	0.462

**Fig. 1:**
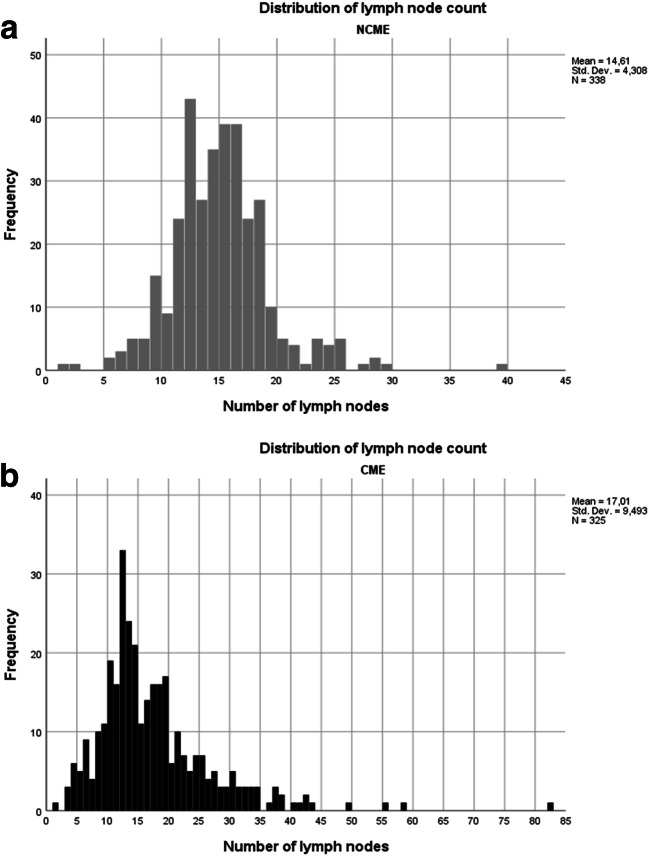
**a** and **b** The number and distribution of harvested lymph nodes

Tumor characteristics and surgical specimen data are shown in Table 2. UICC tumor stage distribution was similar between the study groups, as was the incidence of more advanced T3–4 tumors (*p* = 0.489)*.* The mean number of lymph nodes examined was greater in the CME group (17.0 vs 14.6, *p* < 0.001). In the CME group, specimens were longer (34 cm vs 25 cm), the shortest longitudinal resection margin was wider (8 cm vs 6 cm), and operative time shorter (139 min vs 153 min). Although more lymph nodes were examined in the CME group, surprisingly more patients had at least 12 lymph nodes examined in the NCME group (74.2% in CME vs 80.4% NCME, *p* < 0.005) (Fig 1a and b).

**Table 2:** Tumor characteristics and surgical specimen

	CME	NCME	*p* value
Tumor size median, cm (range)	4.5 (0.2–12.0)	4.5 (0.3–16.0)	0.715
Depth of invasion			0.043
T1	30 (9.3%)	17 (5.0%)	
T2	49 (15.1%)	59 (17.4%)	
T3	205 (63.3%)	204 (60.0%)	
T4	40 (12.3%)	60 (17.6%)	
Histological grade			< 0.001
I	68 (21.9%)	14 (4.2%)	
II	172 (55.3%)	275 (81.6%)	
III	66 (21.2%)	43 (12.8%)	
IV	5 (1.6%)	5 (1.5%)	
Specimen length median, cm (range)	30.0 (13.0–127.0)	23.0 (9.0–85.0)	< 0.001
Shortest longitudinal distance to tumor median, cm (range)	8.0 (1.0–32.0)	6.0 (0.7–25.0)	< 0.001
Number of harvested lymph nodes mean (range)	17.0 (1–82)	14.60 (1–39)	<0.001
Positive lymph nodes mean (range)	1.4 (0–15)	1.0 (0–13)	0.040
> 12 lymph nodes examined Nb (%)	241 (74.2%)	273 (80.3%)	0.048
Lymph node status Nb (%)			0.068
N0	200 (61.5%)	219 (64.4%)	
N1	79 (24.3%)	92 (27.1%)	
N2	46 (14.2%)	29 (8.5%)	
Stage Nb (%)			0.625
I	63 (19.4%)	62 (18.2%)	
II	137(42.2%)	156 (45.9%)	
III	125 (38.5%)	122 (35.9%)

### Survival

**Fig. 2:**
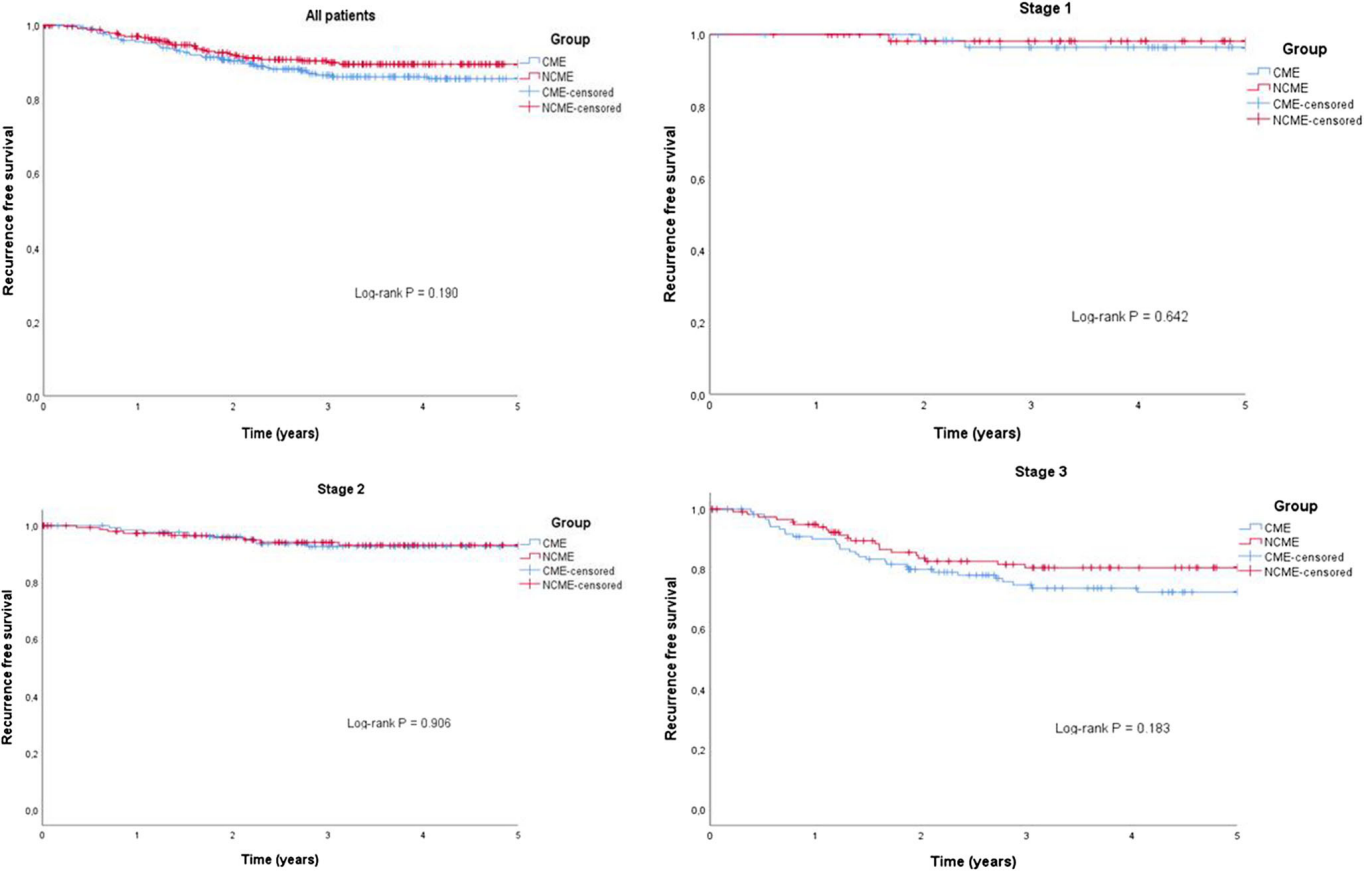
Kaplan–Meier plots of 5-year recurrence-free survival. **a** All patients, **b** stage I patients, **c** stage II patients, **d** stage III patients. CME indicates complete mesocolic excision and NCME noncomplete mesocolic excision

Median follow-up time was 4.4 (IQR 2.7 to 7.2) and 3.9 (IQR 2.2 to 5.7) years in the CME group and NCME groups, respectively. Five-year overall and cancer-specific survivals were similar in both study groups (Table 3). In stage III patients, 5-year cancer-specific survival was similar in both groups with regard to N1 and N2 stage (data not shown). Increasing LNR in stage III patients was associated with poorer survival. Surprisingly, in patients with LNR < 10% DFS was better in the NMCE group than the CME group (97.1% vs 76.9%, respectively, *p* = 0.016). Five-year recurrence-free survival (Fig 2) and locoregional recurrence rate were similar in the CME group and the NCME group (stage I 0% vs 0%, stage II 2.2% vs 1.9%, and stage III 8.8% vs 5.8%, respectively). Right and left side colectomies were also analyzed separately, and there was no significant difference between groups (data not shown). Conversion to open surgery worsened the 5-year cancer-specific survival in the CME group but not in the NCME group (CME group 92.5 to 72.9%, *p* < 0.001; NCME group 93.9 to 86.7%, *p* = 0.098).

**Table 3:** Overall and cancer-specific 5-year survival

Censored							
		Group	Total *N*	*N* of events	*N*	Percent (%)	*p* value
Overall survival							
All patients		CME	325	88	237	72.9	0.528
		NCME	340	75	265	77.9	
Stage	I	CME	63	14	49	77.8	0.675
		NCME	62	10	52	83.9	
	II	CME	137	33	104	75.9	0.685
		NCME	156	34	122	78.2	
	III	CME	125	41	84	67.2	0.262
		NCME	122	31	91	74.6	
Cancer-specific survival							
All patients		CME	325	36	289	88.9	0.132
		NCME	340	23	317	93.2	
Stage	I	CME	63	1	62	98.4	0.907
		NCME	62	1	61	98.4	
	II	CME	137	8	129	94.2	0.892
		NCME	156	7	149	95.5	
	III	CME	125	27	98	78.4	0.072
		NCME	122	15	107	87.7

### Short-Term Outcome

Short-term outcomes are shown in Table 4. The operation time was shorter in the CME group. Conversion to open surgery was done in 59 patients (18.2%) in the CME group vs 30 patients (8.8%) in the NCME group (*p* < 0.001). Postoperative morbidity, 30-day mortality, anastomotic leakage, wound dehiscence, postoperative bleeding, and postoperative hospital stay were similar in both groups. There was no statistical difference (*p* = 0.419) in Clavien–Dindo classification in between the hospitals. There was no statistical difference (*p* = 0.511) in reasons to convert adhesions 30% (NCME) vs 30% (CME), fixed/bulky tumor 30% (NCME) vs 30% (CME), problems with anatomy/exposure 20% (NCME) vs 27% (CME), visceral fat 10% (NCME) vs 10% (CME), and miscellaneous (carcinosis, dilated bowel, hemostasis, etc.).

**Table 4:** Operative data and short-term recovery. *CME*, complete mesocolic excision; *NCME*, noncomplete mesocolic excision

	CME	NCME	*p* value
Operative time minutes, median (range)	135 (58–378)	147 (54–363)	< 0.001
Conversion to open	59 (18.2%)	30 (8.8%)	< 0.001
Hospital stay (days, median)	5.0 (1–62)	5.0 (1–36)	0.878
Overall morbidity	91 (28.0%)	89 (26.2%)	0.597
Surgical	71 (21.8%)	59 (17.4%)	0.340
Leakage	25 (7.7%)	17 (5.0%)	
Bleeding (deep)	9 (2.8%)	5 (1.5%)	
Infection (deep)	4 (1.2%)	2 (0.6%)	
Prolonged postop. ileus	19 (5.8%)	22 (6.5%)	
Wound dehiscence	5 (1.5%)	2 (0.6%)	
Diarrhea	2 (0.6%)	1 (0.3%)	
General	12 (3.7%)	34 (10%)	0.020
Cardiac	3 (0.9%)	5 (1.5%)	
Pulmonary	6 (1.8%)	15 (4.4%)	
Urinary	1 (0.3%)	6 (1.8%)	
Mortality (< 30 days)	5 (1.5%)	5 (1.5%)	0.943

## Discussion

To our knowledge, this is the largest study with the longest follow-up comparing laparoscopic NCME with laparoscopic CME. The results are consistent with earlier studies in that CME provided longer specimens, larger longitudinal margins, and more lymph nodes. Surprisingly in our study, diligently performed laparoscopic NCME colectomy resulted in overall, cancer-specific, and recurrence-free survival rates and locoregional recurrence rates similar to those with laparoscopic CME but did not reduce morbidity or mortality compared with CME. Quality of surgery in our study seems to be reasonably good since both short-term recovery and long-term oncological results in our study are comparable with, or better than, those in CME studies reported by Hohenberger ^6^, Merkel,^7^ and Bertelsen.^10^ There were more conversions (18.2%) in the CME group. Conversion to open surgery has correlated with poorer oncological result in several reports ^19^ as well as in our study.

A number of previous studies and reviews have supported the view that CME results in improved oncologic outcome ^5–8, 11, 12, 20^ but level 1 evidence for CME is missing. The systematic review by Alhassan ^21^ found that only three ^7, 10, 22^ out of six studies demonstrated significantly better DFS in patients operated on by CME. We found similar 5-year overall, cancer-specific, and disease-free survival rates in both study groups in line with a systematic review ^23^ that does not support the use of the CME technique. Detailed analyses of stage III patients according to N1 and N2 lymph node groups and lymph node ratio (LNR) groups (< 10%, 10–25%, and > 25%) showed the CME technique did not result in improved survival.

Only one prospective study comparing CME and NCME has been published (Gao et al. ^24^). In this study, 3-year overall, disease-free, and metastasis-free survival results were similar in both CME and NCME groups. Only local recurrence-free survival was better in the CME group. Of note is that all patients were operated on using the open technique, the number of patients was small and the follow-up short.

Series including historical controls ^6, 7^ may contain bias, because not only surgery but also overall perioperative care such as ERAS protocol and adjuvant chemotherapy has changed over time. On the other hand, studies where dedicated expert surgeons in one hospital performing CME are compared with a heterogeneous group of surgeons in several hospitals performing NCME may have several confounding factors. In a retrospective multicenter study,^10^ CME somewhat surprisingly improved survival in stage I–II patients but not in stage III patients compared with a NCME group. The level of expertise of surgeons performing NCME resections was not described in that study and neither was operative technique in the NCME group. Compared with that study, our follow-up time was longer and the number of patients at risk at the end of the follow-up period was substantially higher, thus strengthening the value of our results. Further, in our study, the expertise of surgeons is well defined in both groups and can be regarded as fairly similar.

Laparoscopy has been advocated as the preferred technique in colon cancer surgery because of short-term advantages and equally good results in terms of long-term cancer survival.^25^ Laparoscopic colectomy is generally performed along Told’s fascia and the mesocolon is dissected medial to lateral respecting embryological planes, as described by Bokey ^4^ and West.^5^ Previous studies have shown that laparoscopic CME is feasible and yields good oncologic results.^26,27^ The detailed technique of CME has been evaluated and reported by two groups of experts.^20,28^ According to Sondenaa et al. ,^20^ there are three key points in CME surgery: dissection between visceral and parietal fascia, central ligation of main vessels, and adequate longitudinal resection of the bowel.

The first key point in CME is to perform the dissection between visceral and parietal fascia. This is not unique to CME but represents common practice in laparoscopic cancer surgery, especially when dissection of the mesocolon is performed medial to lateral. In this respect, both CFCH and PHCH meet the standard of CME. The second key point with CME is central vascular ligation. Lymphatic drainage generally follows arteries, although the direction of flow can be altered in advanced cases. Unlike D2 dissection, CME also removes D3 lymph nodes, but in our study, this did not seem to affect survival. High ligation of IMA was routinely performed in left-sided tumors in both groups.

The third key point is resecting an adequate length of bowel (10 cm from the tumor longitudinally). Metastatic lymph nodes can be detected within 5 to 10 cm of the tumor edge in up to 18% of cases.^29^ Extramesenteric metastatic deposits can be found in 1.1–3.8% of infrapyloric nodes and in 4% of epiploic nodes.^20^ These nodes are removed selectively according to Hohenberger et al., but were not routinely harvested by either group in this study.

### Limitations of this Study

The findings of this study should be interpreted with some caution. The main limitation is the retrospective design and lack of randomization, which may have caused some selection bias. Patient demographics, however, were remarkably similar and both study groups represent contemporary multimodal management of colon cancer. Because of the small number of patients, our study may be underpowered to detect true differences in oncologic outcome between subgroups. The handling of the surgical specimen was not standardized between the two hospitals and was not done according to the principles described by West et al. ^5^. Although the NCME technique used at the time of data acquisition cannot be considered to be true CME as described by Hohenberger and Bertelsen or Japanese D3 level dissection, the quality of NCME and CME surgery in this study seems to be as good as elsewhere, with excellent survival rates.

## Conclusion

Based on our results, we conclude that diligently performed laparoscopic NCME results in similar oncological outcome, morbidity, and mortality compared with the more radical CME technique. The critical elements of CME need more evaluation.
